# Bibliometric analysis of cardiometabolic disorders studies involving NO_2_, PM_2.5_ and noise exposure

**DOI:** 10.1186/s12889-019-7195-1

**Published:** 2019-07-04

**Authors:** Yu-Kai Huang, Rosie Hanneke, Rachael M. Jones

**Affiliations:** 1 School of Public Health, University of Illinois at Chicago, Chicago, USA; 2 Library of the Health Sciences, University of Illinois at Chicago, Chicago, USA

**Keywords:** Bibliometric, Fine particulate matter, PM2.5, Nitrogen dioxide, Multiple, Exposures, Cardiovascular disease, Diabetes, Noise, Cardiometabolic disorders, Study design

## Abstract

**Background:**

This study uses bibliometric analysis to describe the state of research about the association of NO_2_, PM_2.5_ and noise exposures – three traffic-related pollutants – with cardiometabolic disorders.

**Methods:**

We retrieved references published 1994–2017 from Scopus and classified references with respect to exposure, health outcome and study design using index keywords. Temporal trend, top cited references, used index keywords and the number of hypothesis testing and non-hypothesis testing study design for each group were identified.

**Results:**

Results show PM_2.5_ is the most frequently studied exposure (47%), followed by both NO_2_ and PM_2.5_ exposure (29%). Only 3% of references considered multiple exposures between NO_2_ and/or PM_2.5_ and noise, and these were published after 2008. While we observed a growing trend in studies with NO_2_ and/or PM_2.5_ and noise and diabetes in the last decade, there is a diminishing trend in studies with noise and diabetes. Different patterns of study designs were found through H/NH ratio, the number of references classified as having a hypothesis (H)-testing design relative to the number of references classified as having a non-hypothesis (NH)-testing design. Studies with NO_2_ and/or PM_2.5_ exposure are more likely to have a H-testing design, while those with noise exposure are more likely to have a NH-testing design, such as cross-sectional study design.

**Conclusions:**

We conclude with three themes about research trends. First, the study of simultaneous exposures to multiple pollutants is a current trend, and likely to continue. Second, the association between traffic-related pollutants and diabetes and metabolic symptoms is an area for growth in research. Third, the transition to the use of H-testing study designs to explore associations between noise and cardiometabolic outcomes may be supported by improved understanding of the mechanism of action, and/or improvements to the accuracy and precision of air pollution and noise exposure assessments for environmental health research.

**Electronic supplementary material:**

The online version of this article (10.1186/s12889-019-7195-1) contains supplementary material, which is available to authorized users.

## Background

Traffic – road, rail and air – emits a complex mixture of environmental pollutants, including fine particulate matter (PM_2.5_), nitrogen dioxide (NO_2_) and noise. The association between traffic-related air pollutants (TRAPs) and cardiovascular disease has been well established over the past 25 years [1–5]; while the association between TRAPs and diabetes has been established in the last decade [6–9]. The impact of traffic-related noise pollution on cardiovascular disease and cardiometabolic disorders is an emerging area of research [10–12]. While these general trends are known to the environmental health community, this study closely examines trends in research about the association between exposures to PM_2.5_ (fine particulate matter with aerodynamic diameter less than or equal to 2.5 μm) [13], NO_2_ and/or noise with cardiometabolic disorders to gain a better understanding of how research has grown and changed. In addition, we explore how authors have used different study designs over time to describe exposure-outcome association between traffic-related pollutants and cardiometabolic outcomes.

The methodological approach used in this study is bibliometric analysis, which is a quantitative method used to characterize the state of scientific research about a topic through analysis of publications. Bibliometric analysis has been applied in the context of environmental health research, including air quality, typically with a focus on identifying patterns among publications. For example, Tarkowski [14] analyzed publications from Europe about environmental health, and identified that the largest number of articles were in the topics of work environment and health, environmental exposures, and environmental illness. Zell et al. [15] investigated global research activity on air pollution and reported that citations have been rising exponentially since 1991, with most publications coming from investigators in the United States, the United Kingdom and Germany. Wang et al. [16] characterized research on the association between particular matter exposure and atherosclerosis and used cluster analysis of Medical Subject Headings (MeSH) to identify eight clusters that he classified into four key topics of research.

## Methods

### Search strategy

Scopus, a reference database maintained by Elsevier Science, was selected for use because it has a slight advantage in coverage of health sciences, medicine and environmental science recent journals over Web of Science or PubMed [17–19]. Scopus, like many other reference databases, has index keywords that use controlled vocabulary (i.e. MeSH from MEDLINE) to describe various dimensions of the study (e.g., the exposure, the health outcome, the study subject, and study design) and supplement author-identified keywords [20].

The search strategy to identify primary studies was organized around five combinations of the three exposures (NO_2_, PM_2.5_, noise, NO_2_ and PM_2.5_, NO_2_ and/or PM_2.5_ and noise) and the three health outcomes of interest (cardiometabolic disorders, cardiovascular disease and diabetes). The metric of PM_2.5_ was selected, rather than PM_10_ or total suspended particles, because PM_2.5_ is the primary metric of particulate matter used in epidemiologic research the past decade [21], and has been causally associated with cardiovascular disease [4]. A series of inclusion and exclusion criteria were applied to identify relevant references. The search strategy is shown in Table 1. For each of the exposures, two to seven search terms were identified based on the specificity and popularity of the exposure. For each of the health outcomes, MeSH and EMTREE terms were identified for associated health endpoints and biological measurements. After piloting several iterations of search strategies, we used the inclusion criterion of “human” and exclusion criterion of “animal” in all fields to include publications with human subjects and exclude those with animal subjects. In addition, we limited the search to original research studies (not reviews) written in English and published from 1994 through 2017. The year 1994 was selected for the start date because it follows publication of the landmark “six cities study” of air pollution and mortality [2], which initiated a boom in air pollution research in that time period [15]. The search was performed on July 22, 2018.

**Table 1:** Search strategy

Connector	Field	Parameter
	Exposure terms	NO2 OR “nitrogen dioxide” OR pm2. OR “fine particulate matter” OR “particulate matter 2.5” OR (noise AND (“db” OR “decibel” OR “sound level”))
AND	MeSH or EMTREE medical terms of cardiometabolic health consequences	sbp OR “systolic blood pressure” OR dbp OR “diastolic blood pressure” OR “pulse pressure” OR “blood pressure” OR hypertension OR cvd OR cardiovascular OR stroke OR ihd OR “ischemic heart disease” OR cerebrovascular OR “myocardial infarction” OR diabetes OR “glycosylated hemoglobin” OR hba1c OR “diabetes mellitus” OR dm OR “fasting insulin” OR “insulin sensitivity” OR “fasting glucose” OR “acute insulin response to glucose”
AND	Publication date	Jan 1994-Dec 2017
AND	Publication type	Journal article
NOT	Publication type	Review
AND	All fields	Human^a^
NOT	All fields	Animal^b^
AND	Language	English

^a^terms such as human, patients, people or adolescent

^b^terms such as dog, ferret, rabbit, or pig

**Fig. 1:**
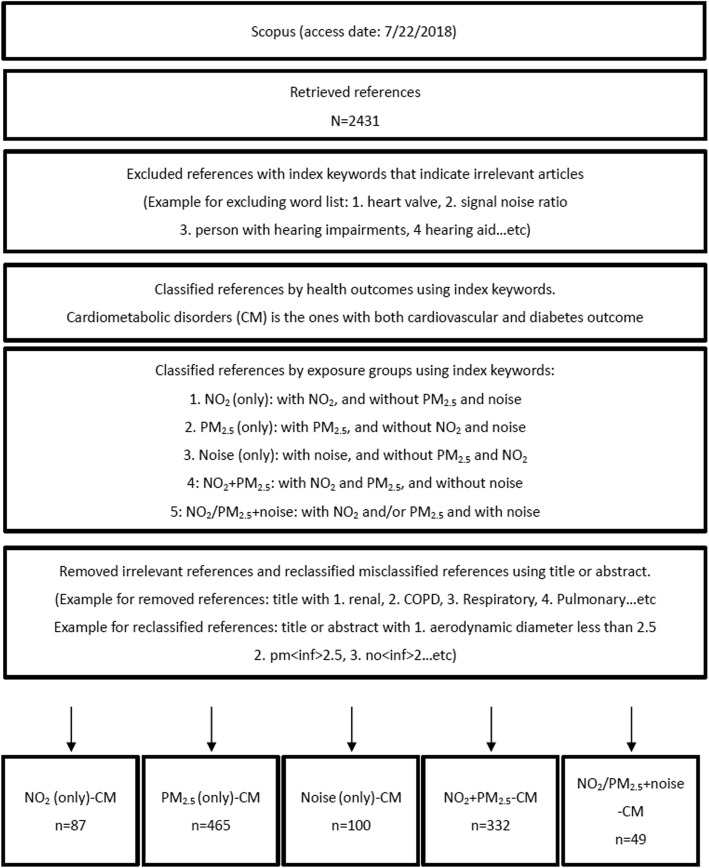
The classification of retrieved references by exposure and health outcome

The retrieved references were then refined using Scopus index keywords and classified. Refinement removed references with index keywords that described an irrelevant topic. For example, references with index keywords like “heart valve” and “signal noise ratio” were in the search results because these terms are associated with studies about the effects of prosthetic valve sound/noise on a cardiovascular patients’ quality of life [22]: These references were removed. Classification labeled each reference with respect to: 1) exposure - NO_2_, PM_2.5_ and/or noise; 2) health outcome - cardiovascular (e.g. stroke, hypertension and cardiovascular disease index terms) and/or diabetes (e.g. diabetes mellitus, glucose blood level and glucose homeostasis index terms); and 3) study design - hypothesis-testing (H-design, index terms: case-control study, retrospective study and longitudinal study) or non-hypothesis-testing (NH-design, index terms: cross-sectional, ecological and health survey). After classification, an additional text review of the title and abstract was performed to verify classification; any errors were corrected. Finally, references were grouped together using different combinations of exposure and/or health outcome classifications for further analysis. Figure 1 describes refinement and classification process.

### Data analysis

Analysis was focused on five groups of references defined using exposure classifications: 1) NO_2_ (only) 2) PM_2.5_ (only) 3) Noise (only) 4) NO_2_ and PM_2.5_ (NO_2_ + PM_2.5_) 5) NO_2_ and/or PM_2.5_ and noise (NO_2_/PM_2.5_ + noise). References in groups 1, 2 and 3 were termed “single exposure” studies because they consider only one of the exposures of interest, while references in groups 4 and 5 were termed “multiple exposures” studies.

For each group of references, we explored temporal trends and tabulated summary statistics, including: number references in the group, published years, and number of citations. We compared the signature index keywords between five groups in two ways: 1) word cloud graphical displays of index keywords, and 2) listing of index keywords appearing in ≥10% of references.

To compare among groups, since the absolute number of references varied substantially, we tabulated the relative frequency of references with different classifications. For example, we tabulated the relative frequency of references that involve each exposure in different time periods: The numerator is the number of references involving the exposure (e.g., NO_2_) in a time period, divided by the total number of references involving the exposure over all time periods.

We defined the H/NH ratio as the ratio of the number of references classified as having a H-design relative to the number of references classified as having a NH-design. Hartwick and Barki [23] described these two study designs as having different purposes: NH-designs are used to search for patterns in data and generate hypotheses, while H-designs are used subsequently to NH-designs to test a specific hypothesis about an association that arose from prior knowledge. This idea suggests that for an exposure-outcome association, NH-designs will be more common than H-designs if the scientific community has not arrived at a consensus opinion about the plausibility or strength of the association, while H-designs are used to examine the proposed association in various circumstances. The H/NH ratio could reveal differences in the state of research among the exposures and outcomes. Differences among groups were tested using the chi-squared test, with the noise (only) exposure group as the reference.

Precision of the reference classification was evaluated by randomly selecting 10% of the references from each of the five exposure groups and the two study design groups, and reviewing the reference abstract and main text to determine if the classification was correct. Any errors identified were corrected before analysis. The data are available in Additional file 7.

## Results

The search process is summarized in Fig. 1. We initially retrieved 2431 references, and identified 1033 references as relevant to NO_2_, PM_2.5_, or noise and to cardiometabolic disorders. Precision of the classification of references was evaluated for 128 references, of which 15 references involved a classification error (Additional file 3: Table S4), for a corrected classification rate of 88%.

**Fig. 2:**
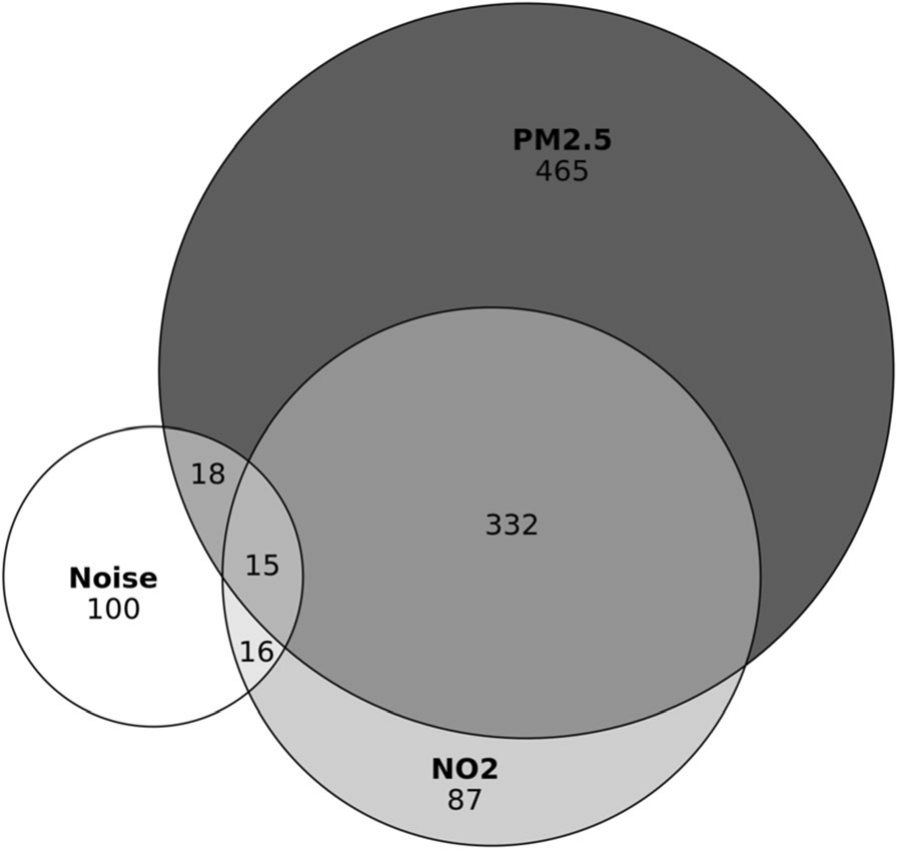
Retrieved references included different combinations of exposures. The numbers indicate the number of retrieved references

**Fig. 3:**
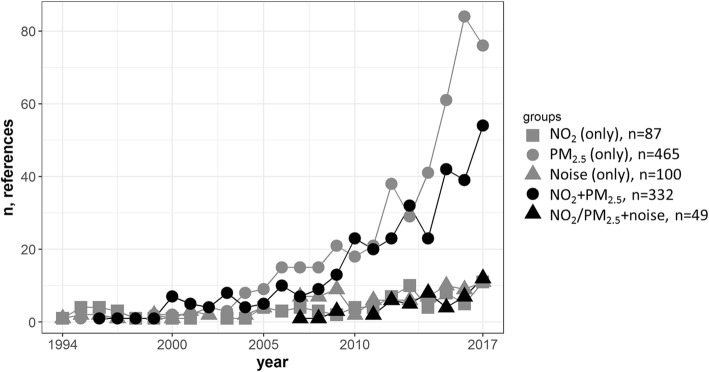
Trend in the number of references about each exposure classification and cardiometabolic disorders

Most of the references are related to cardiovascular disease (*n* = 817, 79%), rather than diabetes (*n* = 100, 10%): Both outcomes were studied in 11% (*n* = 116) of references (see Additional file 1: Figure S1). Figure 2 shows that PM_2.5_ exposure (without NO_2_ and/or noise) is the most frequently studied exposure (*n* = 465, 45%). While many references considered exposures to both PM_2.5_ and NO_2_ (*n* = 332, 32%), few references considered multiple exposures with noise (*n* = 38, 3%). The number of references involving NO_2_ or noise as single exposures are similar (*n* = 87 and *n* = 100, respectively). The temporal trend of the annual number of references is strongly positive for references involving exposures to PM_2.5_ and PM_2.5_ + NO_2_, while the trend is more modest for the other exposure classifications (Fig. 3).

The number of references and citations are summarized by combination of exposure and health outcome classifications in Table 2. For the cardiometabolic outcome, references involving PM_2.5_ exposure were more numerous and were more frequently cited (in total and on average) than other exposure classifications. This combination also had the highest level of skewness in the number of citations per reference, as indicated by the mean number of citations being much larger than the median number of citations (55.5 versus 18 citations). References involving multiple exposures and cardiometabolic (and cardiovascular) outcomes, particularly NO_2_/PM_2.5_ + noise, started to appear in the literature at a later date than references involving single exposures with the same health outcome (Table 2). This is also apparent in Fig. 3. References about diabetes, particularly those with noise exposure, appear in the literature at a later date than references about cardiovascular disease, and are less numerous, but this research area has grown since 2008 (Additional file 2: Figure S2).

**Table 2:** The characteristics of five references groups

Exposure	Health Outcome	N	Publication Year		Times Cited		
			Earliest	Median	Total	Mean	Median
NO_2_ (only)	Cardiometabolic	87	1994	2012	2724	34.1	24
	Cardiovascular	82	1994	2012	2384	31.8	23
	Diabetes	13	1997	2012	503	45.7	27
PM_2.5_ (only)	Cardiometabolic	465	1995	2014	24,713	55.0	18
	Cardiovascular	420	1995	2014	22,712	56.1	17
	Diabetes	109	2001	2015	5789	54.1	23
Noise (only)	Cardiometabolic	100	1994	2011	3658	40.2	18
	Cardiovascular	94	1994	2011	3584	41.2	21
	Diabetes	12	2007	2014.5	174	19.3	13
NO_2_ + PM_2.5_	Cardiometabolic	332	1996	2013	14,935	47.4	20
	Cardiovascular	296	1996	2013	13,969	49.9	21.5
	Diabetes	41	2000	2015	2673	43.1	17
NO_2_/PM_2.5_ + noise	Cardiometabolic	49	2007	2014	1532	31.3	20
	Cardiovascular	41	2007	2014	1310	32.0	22
	Diabetes	18	2008	2016	441	24.5	16.5

**Fig. 4:**
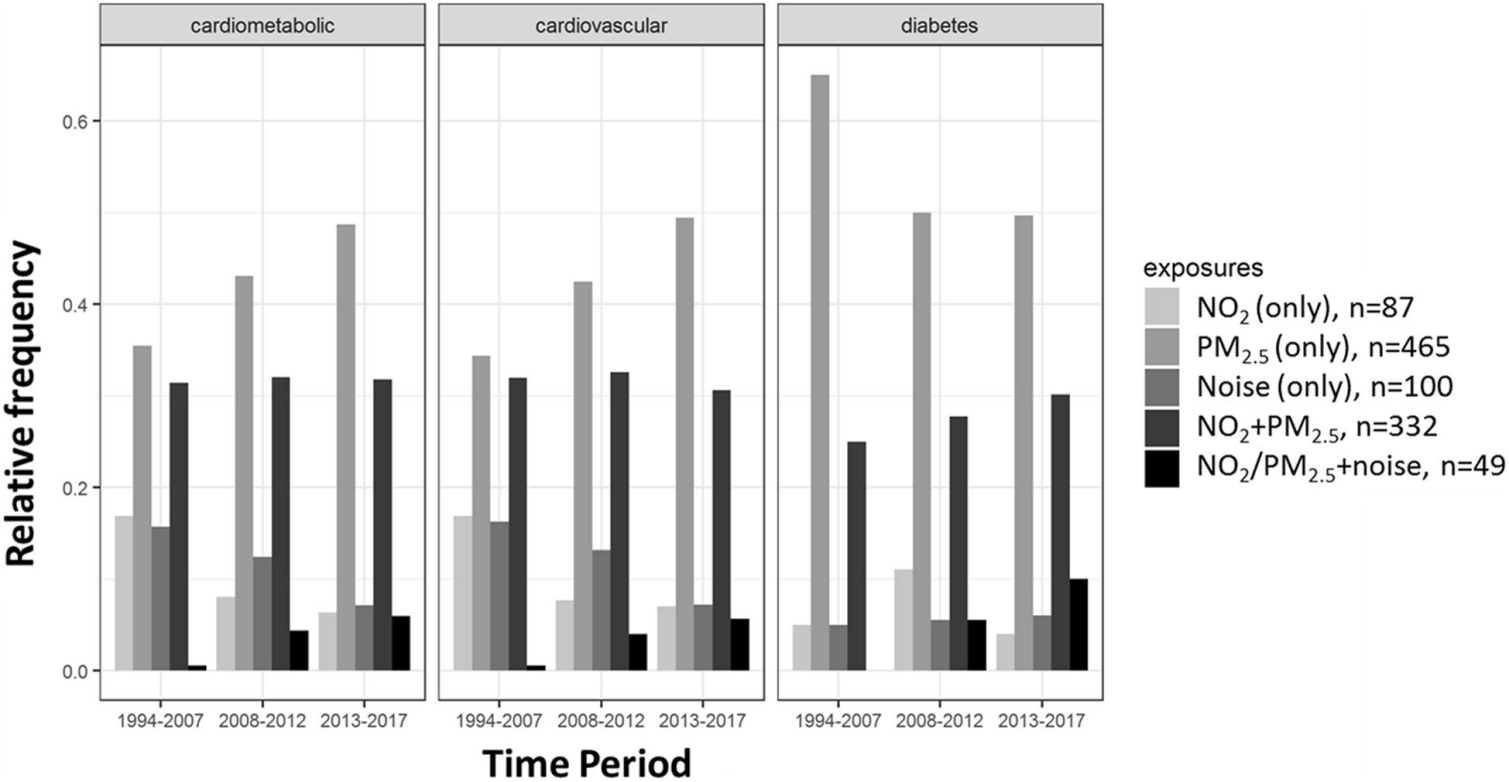
Relative frequency of references studying different exposures by health outcome across three time periods. Relative frequencies (%) in this figure are calculated by the number of total reference involving particular kind of exposure and health outcome divided by the number of total reference involving particular kind of health outcome in that time periods

Figure 4 shows the relative frequency of references involving the different exposure classifications in three periods of time (past 5 years, past 6–10 years, and past 11+ years) for the three health outcomes studied. While the relative frequency of references involving PM_2.5_ and cardiovascular disease has increased over years, the relative frequency of references involving single exposures of NO_2_ and noise have decreased. The number and relative frequency of studies involving multiple exposures with noise (NO_2_/PM_2.5_+ noise) have increased since 2008 for all health outcomes (Table 3 and Fig. 4). The relative frequency of references involving NO_2_ + PM_2.5_ has been steady for cardiovascular disease, but has increased slightly over time for diabetes.

**Table 3:** The top 10 cited references in five references groups

Author^a^	Title	Publish year	Publish journal	Times cited
*NO*_*2*_ *(only)*				
Zmirou D. et al.	Time-series analysis of air pollution and cause-specific mortality	1998	Epidemiology	167
Rich D.Q., Zhu T., Zhang J.J. et al.	Association between changes in air pollution levels during the Beijing Olympics and biomarkers of inflammation and thrombosis in healthy young adults	2012	JAMA	152
Ruidavets J.-B. et al.	Ozone air pollution is associated with acute myocardial infarction	2005	Circulation	146
Brook R.D. et al.	The relationship between diabetes mellitus and traffic-related air pollution	2008	Journal of Occupational and Environmental Medicine	144
Andersen Z.J. et al.	Chronic obstructive pulmonary disease and long-term exposure to traffic-related air pollution: A cohort study	2011	American Journal of Respiratory and Critical Care Medicine	136
Lanki T. et al.	Associations of traffic related air pollutants with hospitalisation for first acute myocardial infarction: the HEAPSS study	2006	Occupational and Environmental Medicine	99
Vedal S. et al.	Air pollution and daily mortality in a city with low levels of pollution.	2003	Environmental health perspectives	85
Henrotin J.B., Giroud M. et al.	Short-term effects of ozone air pollution on ischaemic stroke occurrence: a case-crossover analysis from a 10-year population-based study in Dijon, France	2007	Occupational and Environmental Medicine	84
Dong G.-H., Qian, Z.M. et al.	Association between long-term air pollution and increased blood pressure and hypertension in china	2013	Hypertension	76
Miller O.I. et al.	Guidelines for the safe administration of inhaled nitric oxide	1994	Archives of Disease in Childhood	73
*PM*_*2.5*_ *(only)*				
Pope III C.A. et al.	Health effects of fine particulate air pollution: lines that connect	2006	Journal of the Air and Waste Management Association	2578
Pope III C.A. et al.	Cardiovascular mortality and long-term exposure to particulate air pollution: epidemiological evidence of general pathophysiological pathways of disease	2004	Circulation	1496
Seaton A. et al.	Particulate air pollution and acute health effects	1995	The Lancet	1396
Miller K.A., Kaufman J.D. et al.	Long-term exposure to air pollution and incidence of cardiovascular events in women	2007	New England Journal of Medicine	970
Jerrett M. et al.	Long-term ozone exposure and mortality	2009	New England Journal of Medicine	537
Brook R.D. et al.	Inhalation of fine particulate air pollution and ozone causes acute arterial vasoconstriction in healthy adults	2002	Circulation	532
Pope III C.A. et al.	Cardiovascular mortality and exposure to airborne fine particulate matter and cigarette smoke shape of the exposure-response relationship	2009	Circulation	361
Liao D., Creason J. et al.	Daily variation of particulate air pollution and poor cardiac autonomic control in the elderly	1999	Environmental Health Perspectives	350
Lepeule J. et al.	Chronic exposure to fine particles and mortality: an extended follow-up of the Harvard six cities study from 1974 to 2009	2012	Environmental Health Perspectives	309
Le Tertre A. et al.	Short-term effects of particulate air pollution on cardiovascular diseases in eight European cities	2002	Journal of Epidemiology and Community Health	302
*Noise (only)*				
van Kempen E.E.M.M. et al.	The association between noise exposure and blood pressure and ischemic heart disease: a meta-analysis	2002	Environmental Health Perspectives	285
Jarup L. et al.	Hypertension and exposure to noise near airports: The HYENA study	2008	Environmental Health Perspectives	261
Bluhm G.L. et al.	Road traffic noise and hypertension	2007	Occupational and Environmental Medicine	174
Babisch W. et al.	Road traffic noise and cardiovascular risk	2008	Noise and Health	162
Beelen R. et al., Brunekreef B. et al.	The joint association of air pollution and noise from road traffic with cardiovascular mortality in a cohort study	2009	Occupational and Environmental Medicine	131
Franssen E.A.M., van Wiechen C.M.A.G. et al.	Aircraft noise around a large international airport and its impact on general health and medication use	2004	Occupational and Environmental Medicine	131
Rosenlund M. et al.	Increased prevalence of hypertension in a population exposed to aircraft noise	2001	Occupational and Environmental Medicine	121
de Kluizenaar Y. et al.	Hypertension and road traffic noise exposure	2007	Journal of Occupational and Environmental Medicine	120
Haralabidis A.S., Katsouyanni, K. et al.	Acute effects of night-time noise exposure on blood pressure in populations living near airports	2008	European Heart Journal	104
Bodin T., Bjork J. et al.	Road traffic noise and hypertension: results from a cross-sectional public health survey in southern Sweden	2009	Environmental Health: A Global Access Science Source	103
*NO*_*2*_ *+ PM*_*2.5*_				
Samet J.M. et al.	Fine particulate air pollution and mortality in 20 U.S. cities, 1987–1994	2000	New England Journal of Medicine	1591
Beelen R. et al.	Effects of long-term exposure to air pollution on natural-cause mortality: an analysis of 22 European cohorts within the multicentre ESCAPE project	2014	The Lancet	344
Chuang K.-J., Chan C.-C. et al.	The effect of urban air pollution on inflammation, oxidative stress, coagulation, and autonomic dysfunction in young adults	2007	American Journal of Respiratory and Critical Care Medicine	321
Beelen R., Brunekreef B. et al.	Long-term effects of traffic-related air pollution on mortality in a Dutch cohort (NLCS-AIR study)	2008	Environmental Health Perspectives	270
Mar T.F. et al.	Associations between air pollution and mortality in Phoenix, 1995–1997	2000	Environmental Health Perspectives	266
Kan H. et al.	Season, sex, age, and education as modifiers of the effects of outdoor air pollution on daily mortality in Shanghai, China: The Public Health and Air Pollution in Asia (PAPA) study	2008	Environmental Health Perspectives	243
Wong C.-M. et al.	Public Health and Air Pollution in Asia (PAPA): A multicity study of short-term effects of air pollution on mortality	2008	Environmental Health Perspectives	225
Anderson H.R. et al.	Air pollution and daily mortality in London: 1987–92	1996	British Medical Journal	217
Metzger K.B., Tolbert, P.E. et al.	Ambient air pollution and cardiovascular emergency department visits	2004	Epidemiology	212
Park S.K. et al.	Effects of air pollution on heart rate variability: The VA normative aging study	2005	Environmental Health Perspectives	202
*NO*_*2*_*/PM*_*2.5*_ *+ noise*				
Gan W.Q., Brauer M. et al.	Association of long-term exposure to community noise and traffic-related air pollution with coronary heart disease mortality	2012	American Journal of Epidemiology	98
Fuks K. et al.	Long-term urban particulate air pollution, traffic noise, and arterial blood pressure	2011	Environmental Health Perspectives	94
Hansell A.L., Elliott P. et al.	Aircraft noise and cardiovascular disease near Heathrow airport in London: small area study	2013	BMJ	83
Schneider A. et al.	Endothelial dysfunction: associations with exposure to ambient fine particles in diabetic individuals	2008	Environmental Health Perspectives	82
Davies H.W. et al.	Correlation between co-exposures to noise and air pollution from traffic sources	2009	Occupational and Environmental Medicine	81
Dratva J. et al.	Transportation noise and blood pressure in a population-based sample of adults	2012	Environmental Health Perspectives	79
van Hee V.C. et al.	Exposure to traffic and left ventricular mass and function the multi-ethnic study of atherosclerosis	2009	American Journal of Respiratory and Critical Care Medicine	76
Kaufman J.D. et al.	Association between air pollution and coronary artery calcification within six metropolitan areas in the USA (the multi-ethnic study of atherosclerosis and air pollution): a longitudinal cohort study	2016	The Lancet	70
Krishnan R.M. et al.	Vascular responses to long- and short-term exposure to fine particulate matter: MESA Air (Multi-Ethnic Study of Atherosclerosis and Air Pollution)	2012	Journal of the American College of Cardiology	62
Raaschou-Nielsen O. et al.	Traffic air pollution and mortality from cardiovascular disease and all causes: a Danish cohort study	2012	Environmental Health	56

^a^only shows the first and corresponding author

The ten references with the highest number of citations in each exposure classification group are shown in Table 3. References involving PM_2.5_ are more highly cited than studies involving NO_2_ and noise. The most frequently cited references involving multiple exposures with noise were all published recently, since 2008, while highly cited references involving single pollutants are older.

**Fig. 5:**
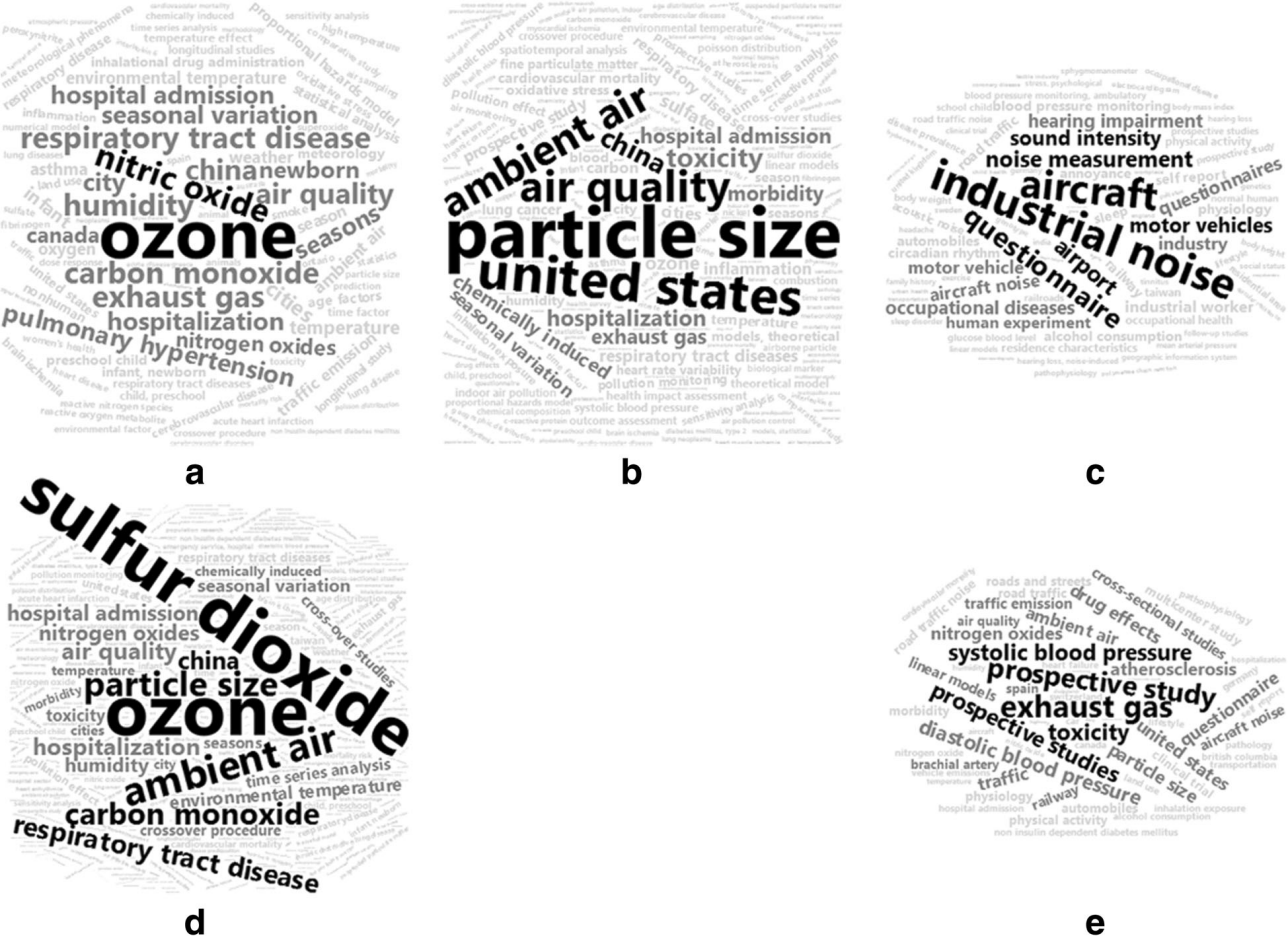
Word clouds for the signature index keywords for references involving (**a**) NO_2_, (**b**) PM_2.5_, (**c**) Noise, (**d**) NO_2_ + PM_2.5_, (**e**) NO_2_/PM_2.5_ + noise. Font size and color of a term is proportional to its relatively frequency and we use black, dark grey and light grey to indicate the relative frequency over 20, 10–20% and less than 10%, respectively

The frequency of signature index keywords for each exposure classification are shown graphically in Fig. 5 and listed in Additional file 4: Table S1. Unsurprisingly, word clouds for the NO_2_, PM_2.5_ and NO_2_ + PM_2.5_ exposure groups are similar, with terms like sulfur dioxide, ozone, particle size, respiratory tract disease and exhaust gas occurred most frequently; followed by terms like, hospitalization, hospital admission, seasonal variation, temperature and air quality. The word cloud for the noise exposure group was different from those for NO_2_ and PM_2.5_, with the terms like aircraft, industrial noise, hearing loss, and questionnaire occurring most frequently; followed by terms like hearing impairment, motor vehicles, and occupational diseases. The word cloud for the NO_2_/PM_2.5_ + noise group looked more like the word cloud for air pollution, but included terms related to noise exposure like aircraft noise and questionnaire.

The number of references classified as H-design and NH-design, and the H/NH ratio are stratified by classifications in Table 4. The H/NH ratios for references involving NO_2_ or PM_2.5_ as single exposures were > 1 for all health outcomes, indicating that more references used an H-design than used a NH-design, but this was not true for references involving noise as a single exposure. This difference in the H/NH ratio was statistically significantly different between references with noise (only) exposure and the others. Additional file 5: Table S2 describes the index keywords for references about noise exposure and cardiometabolic outcomes with H-designs and NH-designs. We found references with H-designs were frequently indexed with the terms “ambient air”, “United States” and “exhaust gas” while references with NH-designs were frequently indexed with the terms “systolic blood pressure”, “diastolic blood pressure” and “motor vehicles”.

**Table 4:** The number of publications with hypothesis-testing designs (H-designs) and non-hypothesis-testing designs (NH-designs), and their ratio by health outcome and exposures

Health Outcome	Exposure	Number of Publications		H/NH ratio	*p*-value (χ^2^)
		H-design	NH-design		
Cardiometabolic	NO_2_ (only)	17	4	4.25	< 0.01
	PM_2.5_ (only)	71	26	2.73	< 0.01
	Noise (only)	11	25	0.44	ref
	NO_2_ + PM_2.5_	46	18	2.56	< 0.01
	NO_2_/PM_2.5_ + noise	14	7	2.00	< 0.01
Cardiovascular	NO_2_ (only)	16	3	5.33	< 0.01
	PM_2.5_ (only)	54	23	2.35	< 0.01
	Noise (only)	9	24	0.38	ref
	NO_2_ + PM_2.5_	38	13	2.92	< 0.01
	NO_2_/PM_2.5_ + noise	10	7	1.43	0.03
Diabetes	NO_2_ (only)	5	1	5.00	0.08
	PM_2.5_ (only)	41	4	10.25	< 0.01
	Noise (only)	2	4	0.50	ref
	NO_2_ + PM_2.5_	11	8	1.34	0.29
	NO_2_/PM_2.5_ + noise	8	1	8.00	0.03

## Discussion

### The state of research

In this study we used bibliometric methods to characterize the state of research about the traffic-related air pollutants NO_2_ and PM_2.5_, noise and cardiometabolic disorders, which included cardiovascular disease and diabetes. Research publications about these topics continue to grow (Fig. 2), and while there is not a shift away from research about cardiovascular disease, there is increased interest in metabolic outcomes, like diabetes. The study of metabolic syndromes and TRAPs began in the early 2000s [24, 25], and was followed by a series of highly-cited references published in the late 2000s in *Environmental Health Perspectives* (Additional file 6: Table S3). Specifically, Ostro et al. [26] and Zanobetti et al. [27] found that diabetes mortality was associated with exposure to PM_2.5_ and Dubowsky et al. [28] and Park et al. [29] found diabetes to modify the effect of air pollution on cardiovascular disease. The cohort Study on the Influence of Air Pollution on Lung Function, Inflammation and Aging (SALIA) was also influential: Using consecutive cross-sectional surveys from 1985 to 1994, the investigators found an association between TRAPs and airway inflammation, diastolic function, and type 2 diabetes incidence [30–32].

PM_2.5_ is a frequently-studied TRAP, particularly since 2000 when Samet et al. [33] and Mar et al. [34] documented an association between particulate matter exposure and cardiovascular mortality. The diminishing frequency of references involving exposure to NO_2_ as a single exposure and cardiovascular disease suggests that the state of knowledge has changed. In particular, as the biological mechanism by which particulate matter impacts cardiovascular disease has been elucidated, it is thought that NO_2_ is not an independent risk factor for cardiovascular disease, and that PM_2.5_ is the TRAPs that causes cardiovascular disease [35, 36].

We observed that the context of research varied among the exposures NO_2_, PM_2.5_ and noise (Fig. 5). For example, references with noise as a single exposure are more likely to have blood pressure as the outcome, while references with other exposures focus on hospital admission or hospitalization. We also observed a location/region difference among the signature index keywords associated with each exposure, which reflects the location of the research institution and/or the geographic setting of the study. For example, the CIBER of Epidemiology and Public Health (CIBERESP) in Spain, has several publications about noise exposure and cardiometabolic disorders, many of which were performed in Spain [37–41], which explains why “Spain” is a signature index keyword. “China” is also a signature index keyword, likely as a result of research by Chinese institutions, but also the global concern about air pollution in China. In a bibliometric analysis of references about atmospheric pollution, Li et al. [42] identified China as one of the most productive countries and that research focused on the characteristics of atmospheric pollution (temporal-spatial distribution and pollutants), rather than health effects of the pollution.

The appearance of certain countries in these results may also be influenced by national environmental standards. For example, while the United States introduced an ambient PM_2.5_ standard in 1997 [43], an ambient PM_2.5_ standard was not introduced by the Republic of Korea until 2015 [44]. While research is not restricted to TRAPs that are the subject of national standards, there are many reasons why researchers would utilize those metrics, including the availability of monitoring data and ability to evaluate health effects relative to regulatory standards. This may be one reason, along with the volume of research, why the United States is a signature index keyword for the PM_2.5_ group.

The trend in the relative frequency of references involving diabetes and single exposure to NO_2_ is not monotonic over the past ten years (Fig. 4), peaking at 12% (2008–2012) before dropping to 4% (2013–2017). In the early 2000s, when research about the association between TRAPs and diabetes was beginning, NO_2_ was easy to measure relative to particulate matter and include in epidemiologic studies [25]. In the 2010s, however, understanding of the pathway by which air pollutants influenced the biological processes of metabolic syndrome and diabetes had improved – e.g., such as the role of chronic inflammation caused by PM_2.5_ exposure [45, 46], which led to a focus on PM_2.5_ rather than NO_2_. By this time, measurements for PM_2.5_ were more common and easier to collect. In addition, there was a shift to the study of multiple exposures rather than single exposures in epidemiologic studies.

The study of simultaneous exposures to multiple pollutants has become a high priority in environmental health research [47]. We found that 37% of references involving NO_2_ or PM_2.5_ included the other exposure. In addition, since 2009, the number of references about noise and cardiometabolic disease has steadily grown (Fig. 3), driven by references that involve noise as one of multiple exposures. Assessing the influence of simultaneous exposures to multiple pollutants is now feasible owing to advances in statistical methods, and the availability of data for exposure assessment. In particular, the majority of the official air sampling sites (such as those operated by the US and Taiwan Environmental Protection Agencies) measure NO_2_ and PM_2.5_, as well as other pollutants. Though we found the relative frequency of references about multiple exposures (NO_2_ + PM_2.5_) and cardiovascular disease have plateaued in the past five years, we expect that research about the impact of the combinations of TRAPs on cardiometabolic disease will increase in the future, due to: continued emphasis on assessing the impact of simultaneous exposures to multiple pollutants [47], interest in other TRAPs like ground level ozone (Fig. 5), improved characterization of particulate matter [48, 49], consideration for TRAPs exposures at locations other than residential address [50, 51], and the increasing use of wearable sensors to measure TRAPs and noise [52–54].

### H-design and NH-design

A paradigm in epidemiologic research is to generate hypotheses about exposure-outcome relationships using study designs that can be implemented relatively rapidly, such as cross-sectional surveys and the use of surveillance data, and then test these hypothesis under a variety of conditions through study designs that enable causal inference, such as a prospective cohort study [55]. In this study, we defined the H/NH ratio to capture the status of an exposure-outcome research along this continuum. Specifically, we posited that exposure-outcome relationships for which H/NH ratio < 1 are newer areas of research, and thus are likely still explored using hypothesis generating study designs. We found that references about the relationship between NO_2_ and/or PM_2.5_ exposure and cardiometabolic disease have H/NH ratio > 1 (Table 4), meaning there are more references using research designs capable of testing the generated hypothesis, and that these references began to appear in the late 1990s and early 2000s (Table 3). In contrast, we found that references about the relationship between noise and cardiometabolic disease have a H/NH ratio < 1 (Table 4), suggesting that hypotheses about this association are still being developed. Research about the association between noise and cardiometabolic disease is not new per se, as noise is an important occupational hazard, but research in environmental noise exposures is expanding (Fig. 5). The oldest reference identified in this study was about the impact of noise on blood pressure among metallurgy workers in 1994 [56], and additional studies would likely have been identified if the time frame of the literature search was changed.

H/NH ratios for references involving multiple exposures with noise are > 1, while H/NH ratios for references involving single exposure to noise are < 0.5. This finding was not necessarily expected owing to the recent grown in research about environmental noise exposure, but may be explained by the addition of noise exposure to epidemiologic studies involving other, more well-established TRAPs, and which, therefore use hypothesis-testing study designs. There are relatively few references with multiple exposures involving noise (*n* = 14), which limits inference from this finding.

The use of index keywords to classify references has some limitations, that particularly affect interpretation of the H/NH ratio. Specifically, the classification of a reference with respect to an exposure does not necessarily mean that it was explored as an independent contributor to the health outcome. For example, Jiménez et al. [57] explored the association between PM_2.5_ and mortality among Spanish elderly, and used noise, gaseous pollutants, tree coverage and temperature as co-variates in their regression model: This reference was classified as PM_2.5_ and noise, but the association between noise and mortality was not specifically explored. Another example is a Danish cross-sectional study by Sørensen et al. [58] in which an association between noise and change in cholesterol identified in a single-exposure model disappeared in a multi-pollutant model, suggesting that the independent effect of noise was small relative to that of NO_2_ or PM_2.5_. These two examples demonstrate why we are conservative about the interpretation of H/NH ratio for references involving multiple exposures with noise. Nevertheless, we think the H/NH ratio is a useful tool for characterizing the stage of establishment of exposure-outcome relationships in research.

### Study limitation

This study focused on three exposures (PM_2.5_, NO_2_ and noise) and cardiometabolic disorders, which is only a subset of epidemiologic research about TRAP. Specifically, this search strategy excluded references about PM_10_, NO_x_ and ozone that did not also address PM_2.5_, NO_2_ or noise. While other air pollutants may be important to environmental health, we focused on PM_2.5_ and NO_2_ because they are representative of the solid and gaseous phase of TRAPs, and they are being targeted in two major European air pollution studies, the European Study of Cohorts of Air Pollution Effects (ESCAPE) [59, 60] and the Effects of Low-Level Air Pollution (ELAPSE) study [61]. However, we do recognize that ozone is an air pollutant of importance to environmental health and we encourage future bibliometric research that considers this, and other air pollutants.

Another limitation of our study is our use of index keywords to classify references with respect to exposure, health outcome and study design as inaccurately assigned keywords could influence our findings. We tried to limit the misclassification by revisiting the classification using text search of the reference title and abstract, not just index keyword. We found that classification was correct for 88% of references evaluated manually, and judged this acceptable given the study objectives. An example of the classification is the reference by Beelen et al. [12], in which the index keywords and abstract used the term “black smoke” to describe the air pollution exposure metric, though the text also examined the associations between PM_2.5_ and NO_2_ and cardiovascular mortality.

## Conclusions

Our analysis identified several themes in current research that are likely to continue in the future. First, the study of simultaneous exposures to multiple pollutants is a current trend, and likely to continue, in part due to the increased availability of low-cost and wearable measurement devices that can assess exposure to multiple pollutants with high spatial and temporal resolution; and can be utilized by an army of citizen scientists [53]. Second, the association between TRAPs and diabetes is a growing area of research, and will likely expand to other metabolic syndromes as we learn more about the biological processes of disease and role of environmental factors. Third, while research involving hypothesis-testing study designs exploring the associations between TRAPs and cardiometabolic outcomes is ongoing, further research appears necessary to facilitate application of these research designs to study the association between noise and cardiometabolic outcomes. This transition may be supported by improved understanding of the mechanism of action, and/or improvements to the accuracy and precision of air pollution and noise exposure assessments for environmental health research.

## Additional files

Additional file 1: **Figure S1.** Retrieved references included different combinations of outcomes. The numbers indicate the number of retrieved references. (TIF 3076 kb)

Additional file 2: **Figure S2.** Relative frequency of references with cardiovascular outcomes and diabetes as studied in three exposure groups across three time periods. Relative frequencies in this figure are calculated by the number of total references involving a particular kind of exposure and health outcome divided by the number of total references involving the particular exposure in that time periods. (TIF 3750 kb)

Additional file 3: **Table S4.** The precision test. (PDF 252 kb)

Additional file 4: **Table S1.** Top 20 signature index keywords for five references groups. (PDF 127 kb)

Additional file 5: **Table S2.** Difference in index keywords between cardiometabolic references involving noise exposure with H-designs and NH-designs. (PDF 86 kb)

Additional file 6: **Table S3.** The top 10 cited references involving diabetes. (PDF 168 kb)

Additional file 7: The data set, including references identified in the literature search and classifications. (CSV 4854 kb)

## Data Availability

All data are contained within the manuscript and its additional files.

## Abbreviations

H-design: Hypothesis-testing study design
MeSH: Medical Subject Headings
NH-design: Non-hypothesis-testing study design
NO_2_: Nitrogen dioxide
PM_10_: Particulate matter with aerodynamic diameter less than or equal to 10 μm
PM_2.5_: Fine particulate matter with aerodynamic diameter less than or equal to 2.5 μm
TRAPs: Traffic-related air pollutants
TSP: Total suspended particles
