# Supplementary figures and images for: Bibliometric analysis of cardiometabolic disorders studies involving NO2, PM2.5 and noise exposure
Source: BMC Public Health. 2019 Jul 4;19:877. doi: 10.1186/s12889-019-7195-1 (PMC6610906; doi:10.1186/s12889-019-7195-1)

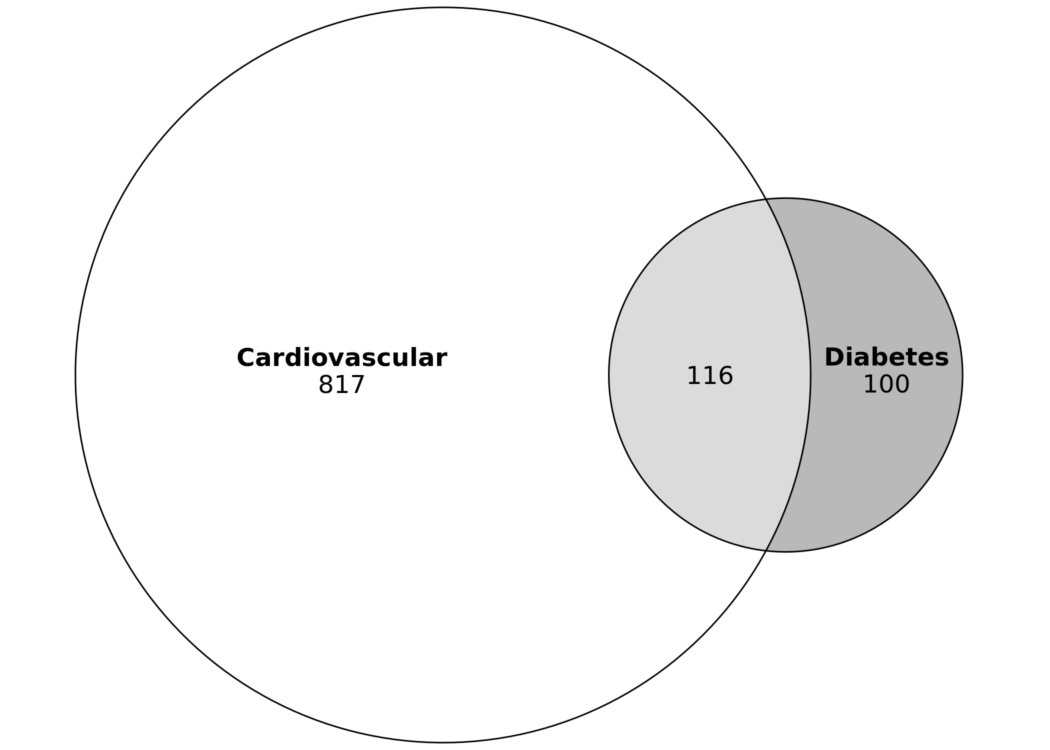

Supplement: Supplementary file 1 — Figure S1. Retrieved references included different combinations of outcomes. The numbers indicate the number of retrieved references. (TIF 3076 kb) [file 12889_2019_7195_MOESM1_ESM.tif]

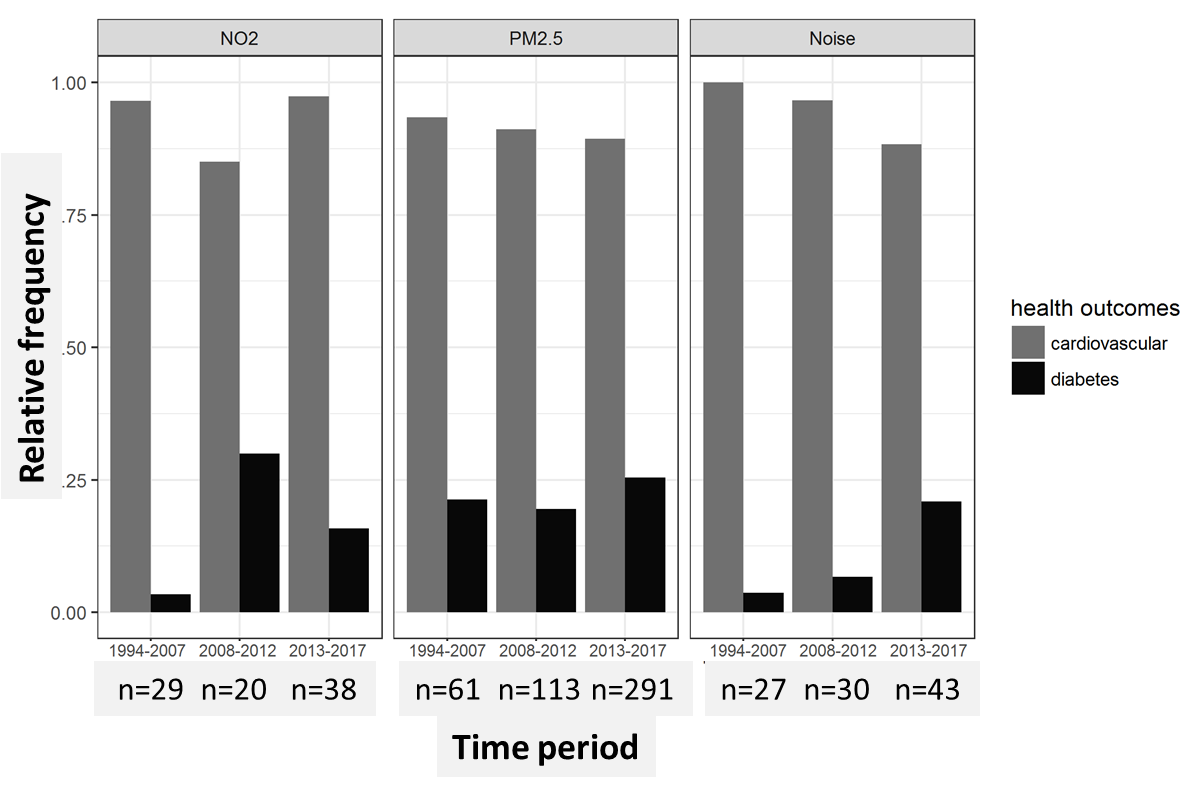

Supplement: Supplementary file 2 — Figure S2. Relative frequency of references with cardiovascular outcomes and diabetes as studied in three exposure groups across three time periods. Relative frequencies in this figure are calculated by the number of total references involving a particular kind of exposure and health outcome divided by the number of total references involving the particular exposure in that time periods. (TIF 3750 kb) [file 12889_2019_7195_MOESM2_ESM.tif]
