# Supplementary material for: Bibliometric analysis of cardiometabolic disorders studies involving NO2, PM2.5 and noise exposure
Source: BMC Public Health. 2019 Jul 4;19:877. doi: 10.1186/s12889-019-7195-1 (PMC6610906; doi:10.1186/s12889-019-7195-1)
Supplement: Supplementary file 3 — Table S4. The precision test. (PDF 252 kb) [file 12889_2019_7195_MOESM3_ESM.pdf]

S4 Table. The precision test

Author*	Title	Publish year	Classification groups$	Incorrect classification
Adams L., Estevez, A.G. et al.	Reactive nitrogen species in cellular signaling	2015	1	
Chen H. et al.	Long-term exposure to traffic-related air pollution and cardiovascular mortality	2013	1	
Coogan P.F. et al.	Long-term exposure to no2 and ozone and hypertension incidence in the black women's health study	2017	1	
Goldberg M.S. et al.	The short-term influence of temperature on daily mortality in the temperate climate of montreal, canada	2011	1	
Karakitsios S., Asikainen, A. et al.	Integrated exposure for risk assessment in indoor environments based on a review of concentration data on airborne chemical pollutants in domestic environments in europe	2015	1	
Lanki T. et al.	Associations of traffic related air pollutants with hospitalisation for first acute myocardial infarction: the heapss study	2006	1	
Lazarevic N., Knibbs, L.D. et al.	Long-term ambient air pollution exposure and self-reported morbidity in the australian longitudinal study on women's health: a cross-sectional study	2015	1	
Theoharatos G., Pantavou, K. et al.	Heat waves observed in 2007 in athens, greece: synoptic conditions, bioclimatological assessment, air quality levels and health effects	2010	1	
Zhang Z., Kan, H. et al.	Impact of haze and air pollution-related hazards on hospital admissions in guangzhou, china	2014	1	
Atkinson R.W. et al.	Fine particle components and health - a systematic review and meta-analysis of epidemiological time series studies of daily mortality and hospital admissions	2015	2	IC
Balluz L. et al.	Ischemic heart disease and ambient air pollution of particulate matter 2.5 in 51 counties in the u.s.	2007	2	

Barman S.C. et al.	Fine particles (pm2.5) in ambient air of lucknow city due to fireworks on diwali festival	2009	2	IC
Brook R.D. et al.	Long-term fine particulate matter exposure and mortality from diabetes in canada	2013	2	
Calderon-Garciduenas L. et al.	Mexico city normal weight children exposed to high concentrations of ambient pm2.5 show high blood leptin and endothelin-1, vitamin d deficiency, and food reward hormone dysregulation versus low pollution controls. Relevance for obesity and alzheimer disease	2015	2	
Chuang K.-J., Chan, C.-C. et al.	Effects of particle size fractions on reducing heart rate variability in cardiac and hypertensive patients	2005	2	
Crouse D.L. et al.	Risk of nonaccidental and cardiovascular mortality in relation to long-term exposure to low concentrations of fine particulate matter: a canadian national-level cohort study	2012	2	
Fang D., Wang, Q et al..	Mortality effects assessment of ambient pm2.5 pollution in the 74 leading cities of china	2016	2	
Galvis B. et al.	Air quality impacts and health-benefit valuation of a low-emission technology for rail yard locomotives in atlanta georgia	2015	2	IC
Haley V.B. et al.	Surveillance of the short-term impact of fine particle air pollution on cardiovascular disease hospitalizations in new york state	2009	2	
Hamilton I. et al.	Health effects of home energy efficiency interventions in england: a modelling study	2015	2	IC
Hart J.E. et al.	The association of long-term exposure to pm2.5 on all-cause mortality in the nurses' health study and the impact of measurement-error correction	2015	2	IC
He F., Liao, D. et al..	Acute effects of fine particulate air pollution on cardiac arrhythmia: the apacr study	2011	2	
Honda T. et al.	Long-term exposure to residential ambient fine and coarse particulate matter and incident hypertension in post-menopausal women	2017	2	
Hu, Z. et al.	Spatial analysis of modis aerosol optical depth, pm2.5, and chronic coronary heart disease	2009	2	
Huang C., Gu, D. et al.	Potential cardiovascular and total mortality benefits of air pollution control in urban china	2017	2	
Janssen N.A.H. et al.	Associations between ambient, personal, and indoor exposure to fine particulate matter	2005	2	

	constituents in dutch and finnish panels of cardiovascular patients			
Kaufman J.D. et al.	Prospective study of particulate air pollution exposures, subclinical atherosclerosis, and clinical cardiovascular disease	2012	2	
Lanzinger S. et al.	Ultrafine and fine particles and hospital admissions in central europe results from the ufireg study	2016	2	
Leem J.H. et al.	Public-health impact of outdoor air pollution for 2nd air pollution management policy in seoul metropolitan area, korea	2015	2	
Lim Y.-H., Hong, Y.-C. et al.	Vascular and cardiac autonomic function and pm2.5 constituents among the elderly: a longitudinal study	2017	2	
Lin H., Qian, Z. et al.	Ambient pm2.5 and stroke: effect modifiers and population attributable risk in six low- and middle-income countries	2017	2	
Mar T.F., Hopke, P.K et al..	Pm source apportionment and health effects. 3. investigation of inter-method variations in associations between estimated source contributions of pm2.5 and daily mortality in phoenix, az	2006	2	
Marshall J.D. et al.	Blue skies bluer?	2015	2	
McGuinn L.A., Devlin, R.B. et al.	Fine particulate matter and cardiovascular disease: comparison of assessment methods for long-term exposure	2017	2	
Pandolfi M. et al.	Effect of atmospheric mixing layer depth variations on urban air quality and daily mortality during saharan dust outbreaks	2014	2	IC
Park S.K. et al.	Particulate air pollution, metabolic syndrome, and heart rate variability: the multi-ethnic study of atherosclerosis (mesa)	2010	2	
Park S.K. et al.	Traffic-related particles are associated with elevated homocysteine: the va normative aging study	2008	2	

Pearson J.F., Brownstein, J. S. et al.	Association between fine particulate matter and diabetes prevalence in the u.s.	2010	2	
Pope C.A. et al.	Relationships between fine particulate air pollution, cardiometabolic disorders, and cardiovascular mortality	2015	2	
Repace J.L. et al.	Fine particle air pollution and secondhand smoke exposures and risks inside 66 us casinos	2011	2	
Rojas-Rueda D. et al.	Health impact assessment of increasing public transport and cycling use in barcelona: a morbidity and burden of disease approach	2013	2	
Solomon P.A. et al.	Air pollution and health: bridging the gap from sources to health outcomes: conference summary	2012	2	IC
Stockfelt L. et al.	Short-term chamber exposure to low doses of two kinds of wood smoke does not induce systemic inflammation, coagulation or oxidative stress in healthy humans	2013	2	
Sullivan J. et al.	Relation between short-term fine-particulate matter exposure and onset of myocardial infarction	2005	2	
Teichert T. et al.	Association between traffic-related air pollution, subclinical inflammation and impaired glucose metabolism: results from the salia study	2013	2	IC
Valdes A. et al.	Elemental concentrations of ambient particles and cause specific mortality in santiago, chile: a time series study	2012	2	
Villeneuve P.J. et al.	Long-term exposure to fine particulate matter air pollution and mortality among canadian women	2015	2	
Wang M., Rich, D.Q et al..	Does total antioxidant capacity modify adverse cardiac responses associated with ambient ultrafine, accumulation mode, and fine particles in patients undergoing cardiac rehabilitation?	2016	2	
Wang Y. et al.	Short-term changes in ambient particulate matter and risk of stroke: a systematic review and	2014	2	

	meta-analysis		
Wang Y.-C., Lin, Y.-K. et al.	Mortality and emergency room visits associated with ambient particulate matter constituents in metropolitan taipei	2016	2
Weichenthal S. et al.	Biomass burning as a source of ambient fine particulate air pollution and acute myocardial infarction	2017	2
White L.F. et al.	Temporal aspects of air pollutant measures in epidemiologic analysis: a simulation study	2016	2
Xia Y., Guan, D. et al.	Assessment of socioeconomic costs to china's air pollution	2016	2
Xu A., Zhang, L. et al.	Acute effects of particulate air pollution on ischemic heart disease hospitalizations in shanghai, china	2017	2
Zanobetti A. et al.	A national case-crossover analysis of the short-term effect of pm2.5 on hospitalizations and mortality in subjects with diabetes and neurological disorders	2014	2
Zhang Q., Cai, Y.-D. et al.	The use of protein-protein interactions for the analysis of the associations between pm2.5 and some diseases	2016	2
Chang T.-Y. et al.	Noise frequency components and the prevalence of hypertension in workers	2012	3
Kankaria A. et al.	Road traffic noise: a risk factor for myocardial infarction?	2013	3
Khaiwal R. et al.	Assessment of noise pollution in and around a sensitive zone in north india and its non-auditory impacts	2016	3
Nawaz S.K., Hasnain, S. et al.	Association of ace id and ace g2350a polymorphism with increased blood pressure in persons exposed to different sound levels in pakistan	2011	3
Ni C.-H. et al.	Associations of blood pressure and arterial compliance with occupational noise exposure in female workers of textile mill	2007	3
Sobotova L. et al.	Community response to environmental noise and the impact on cardiovascular risk score	2010	3
Sorensen M. et al.	Exposure to road traffic and railway noise and associations with blood pressure and	2011	3

	self-reported hypertension: a cohort study			
Tong J., Yuan, J. et al.	Effect of interaction between noise and a1166c site of at1r gene polymorphism on essential hypertension in an iron and steel enterprise workers	2017	3	
Van Helleputte N. et al.	A 160 a biopotential acquisition ic with fully integrated ia and motion artifact suppression	2012	3	IC
Virkkunen H. et al.	Long-term effect of occupational noise on the risk of coronary heart disease	2005	3	
Agay-Shay K. et al.	Air pollution and congenital heart defects	2013	4	
Basu R. et al.	The effect of high ambient temperature on emergency room visits	2012	4	
Bentayeb M. et al.	Association between long-term exposure to air pollution and mortality in france: a 25-year follow-up study	2015	4	
Bilenko N., Gehring, U. et al.	Associations between particulate matter composition and childhood blood pressure - the piama study	2015	4	
Chen L.	The air quality health index as a predictor of emergency department visits for ischemic stroke in edmonton, canada	2014	4	
Chen Y.-C., Yang, C.-Y. et al.	Short-term effects of coarse particulate matter on hospital admissions for cardiovascular diseases: a case-crossover study in a tropical city	2015	4	
Cheng H.-C., Wang, A.-G. et al.	Ambient air pollution and the risk of central retinal artery occlusion	2016	4	
Chiu H.-F., Yang, C.-Y. et al.	Short-term effects of fine particulate air pollution on ischemic stroke occurrence: a case-crossover study	2013	4	
Chuang K.-J., Cheng, T.-J. et al.	Long-term air pollution exposure and risk factors for cardiovascular diseases among the elderly in taiwan	2011	4	
Collart P. et al.	Short-term effects of air pollution on hospitalization for acute myocardial infarction: age effect on lag pattern	2017	4	IC

DeVries R. et al.	Low level air pollution and exacerbation of existing copd: a case crossover analysis	2016	4	IC
Eze I.C. et al.	Long-term exposure to ambient air pollution and metabolic syndrome in adults	2015	4	
Goldberg M.S. et al.	Associations between ambient air pollution and daily mortality among elderly persons in montreal, quebec	2013	4	
Gurjar B.R. et al.	Human health risks in megacities due to air pollution	2010	4	IC
Holguin F. et al.	Air pollution and heart rate variability among the elderly in mexico city	2003	4	
Jafta N. et al.	Indoor air quality of low and middle income urban households in durban, south africa	2017	4	
Johnson J.Y., Villeneuve, P.J. et al.	A retrospective cohort study of stroke onset: implications for characterizing short term effects from ambient air pollution	2011	4	
Kim, H. et al.	Effect of asian dust storms on daily mortality in seven metropolitan cities of korea	2013	4	
Lenters V. et al.	Long-term exposure to air pollution and vascular damage in young adults	2010	4	
Liu R., Li, T. et al.	The relationship between airborne fine particle matter and emergency ambulance dispatches in a southwestern city in chengdu, china	2017	4	
Madhloum N., Nawrot, T.S. et al.	Cord plasma insulin and in utero exposure to ambient air pollution	2017	4	
Mate T., Diaz, J. et al.	Short-term effect of fine particulate matter (pm2.5) on daily mortality due to diseases of the circulatory system in madrid (spain)	2010	4	
Mechtouff L. et al.	Lack of association between air pollutant exposure and short-term risk of ischaemic stroke in lyon, france	2012	4	
Mobasher Z., Wilson, M.L. et al.	Associations between ambient air pollution and hypertensive disorders of pregnancy	2013	4	
Naess O. et al.	Relation between concentration of air pollution and cause-specific mortality: four-year exposures to nitrogen dioxide and particulate matter pollutants in 470 neighborhoods in	2007	4	

	oslo, norway			
Qiu H. et al.	Differential effects of fine and coarse particles on daily emergency cardiovascular hospitalizations in hong kong	2013	4	
Shutt R.H. et al.	Exposure to air pollution near a steel plant is associated with reduced heart rate variability: a randomised crossover study	2017	4	
Szyszkowicz M. et al.	Even low levels of ambient air pollutants are associated with increased emergency department visits for hypertension	2012	4	IC
Tuan T.S., Nascimento, L.F.C. et al.	Effects of air pollutant exposure on acute myocardial infarction, according to gender	2016	4	
Weichenthal S. et al.	Ambient pm2.5 and risk of emergency room visits for myocardial infarction: impact of regional pm2.5 oxidative potential: a case-crossover study	2016	4	
Wellenius G.A. et al.	Effects of ambient air pollution on functional status in patients with chronic congestive heart failure: a repeated-measures study	2007	4	
Yang H.-C., Liou, D.-M. et al.	The effect of particulate matter size on cardiovascular health in taipei basin, taiwan	2016	4	
Zanoli L. et al.	A systematic review of arterial stiffness, wave reflection and air pollution	2017	4	
Dales R. et al.	Particulate air pollution and vascular reactivity: the bus stop study	2007	5	
Eze I.C. et al.	Long-term exposure to transportation noise and air pollution in relation to incident diabetes in the sapaldia study	2017	5	
Foraster M. et al.	Exposure to road, railway, and aircraft noise and arterial stiffness in the sapaldia study: annual average noise levels and temporal noise characteristics	2017	5	
Pedersen M. et al.	Gestational diabetes mellitus and exposure to ambient air pollution and road traffic noise: a cohort study	2017	5	

Gan W.Q., Brauer, M. et al.	Association of long-term exposure to community noise and traffic-related air pollution with coronary heart disease mortality	2012	5	
Eze I.C. et al.	Exposure to night-time traffic noise, melatonin-regulating gene variants and change in glycemia in adults	2017	H	
Rich, D.Q. et al.	Ambient fine particulate air pollution triggers st-elevation myocardial infarction, but not non-st elevation myocardial infarction: a case-crossover study	2014	H	
Grazuleviciene R. et al.	Exposure to urban nitrogen dioxide pollution and the risk of myocardial infarction	2004	H	
Green R., Broadwin, R. et al.	Long- and short-term exposure to air pollution and inflammatory/hemostatic markers in midlife women	2016	H	
Kan, H. et al.	Associations between long-term exposure to ambient particulate air pollution and type 2 diabetes prevalence, blood glucose and glycosylated hemoglobin levels in china	2016	H	IC
Lu M.-C., Yan, Y.-H. et al.	Association of temporal distribution of fine particulate matter with glucose homeostasis during pregnancy in women of chiayi city, taiwan	2017	H	
Peng C. et al.	Associations of annual ambient fine particulate matter mass and components with mitochondrial dna abundance	2017	H	
Ranzi A., Forastiere, F. et al.	Mortality and morbidity among people living close to incinerators: a cohort study based on dispersion modeling for exposure assessment	2011	H	
Rodosthenous R.S. et al.	Ambient particulate matter and micrornas in extracellular vesicles: a pilot study of older individuals	2016	H	
Ruckerl R. et al.	Air pollution and inflammation (interleukin-6, c-reactive protein, fibrinogen) in myocardial infarction survivors	2007	H	
Thurston G.D. et al.	Ambient particulate matter air pollution exposure and mortality in the nih-aarp diet and health cohort	2016	H	

To T. et al.	Chronic disease prevalence in women and air pollution - a 30-year longitudinal cohort study	2015	H	
Wellenius, G. A. et al.	Ambient air pollution and depressive symptoms in older adults: results from the mobilize boston study	2014	H	
Wellenius G.A. et al.	Ambient particulate matter and the response to orthostatic challenge in the elderly: the maintenance of balance, independent living, intellect, and zest in the elderly (mobilize) of boston study	2012	H	
Zanobetti A. et al.	Associations between arrhythmia episodes and temporally and spatially resolved black carbon and particulate matter in elderly patients	2014	H	
Zhang Z., Hart, J. et al.	Long-term exposure to particulate matter and self-reported hypertension: a prospective analysis in the nurses??Health study	2016	H	
Chiusolo M., Berti, G. et al.	Short-term effects of nitrogen dioxide on mortality and susceptibility factors in 10 italian cities: the epiair study	2011	NH	IC
Gupta, G. et al.	Metabolic syndrome in female workers from a textile mill: effect of noise	2017	NH	
Liu C., Kan, H. et al.	Associations between ambient fine particulate air pollution and hypertension: a nationwide cross-sectional study in china	2017	NH	
Maheswaran R. et al.	Outdoor air pollution and incidence of ischemic and hemorrhagic stroke: a small-area level ecological study	2012	NH	
Nawaz S.K. et al.	Effect of ace polymorphisms on the association between noise and hypertension in a pakistani population	2011	NH	
Pollard S.L., Checkley, W. et al.	A cross-sectional study of determinants of indoor environmental exposures in households with and without chronic exposure to biomass fuel smoke	2014	NH	
Teichert T. et al.	Association between traffic-related air pollution, subclinical inflammation and impaired glucose metabolism: results from the salia study	2013	NH	

Yang C.-M. et al.	Reducing fine particulate to improve health: a health impact assessment for taiwan	2013	NH

*only shows the first and corresponding author

$classification group: 1) NO_2 (only) 2) $PM_{2.5}$ (only) 3) noise (only) 4) NO_2 and $PM_{2.5}$ (NO_2+$PM_{2.5}$) 5) NO_2 and/or $PM_{2.5}$ and noise (NO_2/$PM_{2.5}$+noise) H) H-design NH) NH-design
