# Supplementary material for: Bibliometric analysis of cardiometabolic disorders studies involving NO2, PM2.5 and noise exposure
Source: BMC Public Health. 2019 Jul 4;19:877. doi: 10.1186/s12889-019-7195-1 (PMC6610906; doi:10.1186/s12889-019-7195-1)
Supplement: Supplementary file 4 — Table S1. Top 20 signature index keywords for five references groups. (PDF 127 kb) [file 12889_2019_7195_MOESM4_ESM.pdf]

S1 Table. Top 20 signature index keywords for five references groups

NO_2 (only)		$PM_{2.5}$ (only)		Noise (only)	
Index keyword	frequency	Index keyword	frequency	Index keyword	frequency
sulfur dioxide	30	particle size	170	traffic noise	37
ozone	29	atmospheric pollution	149	noise, transportation	31
atmospheric pollution	17	united states	127	noise, occupational	29
nitric oxide	14	air quality	104	industrial noise	23
carbon monoxide	13	ambient air	101	diastolic blood pressure	23
exhaust gas	13	respiratory tract disease	89	systolic blood pressure	22
humidity	13	china	65	aircraft	21
respiratory tract disease	13	toxicity	64	cross-sectional studies	20
china	12	hospitalization	54	blood pressure measurement	16
air quality	12	hospital admission	52	questionnaire	15
hospitalization	11	exhaust gas	50	airport	12
pulmonary hypertension	11	morbidity	49	noise measurement	12
seasonal variation	11	chemically induced	47	motor vehicles	11
city	11	seasonal variation	46	sound intensity	11
hospital admission	11	ozone	44	questionnaires	10
seasons	11	sulfate	44	hearing impairment	10
newborn	10	inflammation	41	occupational diseases	10
canada	9	chronic obstructive lung disease	41	aircraft noise	9
nitrogen oxides	9	respiratory tract diseases	40	industry	9
cities	9	cities	39	motor vehicle	9

NO_2+$PM_{2.5}$		$NO_{2/}PM_{2.5}$+noise	
Index keyword	frequency	Index keyword	frequency
ozone	186	traffic noise	28
sulfur dioxide	184	noise, transportation	20
atmospheric pollution	121	atmospheric pollution	15
ambient air	107	exhaust gas	12
particle size	91	prospective study	11
carbon monoxide	80	prospective studies	9
respiratory tract disease	73	systolic blood pressure	9
china	65	toxicity	9
hospitalization	63	diastolic blood pressure	8
air quality	62	united states	7
nitrogen oxides	58	atherosclerosis	7
hospital admission	57	particle size	7
humidity	52	ambient air	7
environmental temperature	47	drug effects	7
seasonal variation	46	nitrogen oxides	7
toxicity	45	questionnaire	7
time series analysis	41	traffic	7
seasons	41	aircraft noise	6
cross-over studies	40	cross-sectional studies	6
crossover procedure	38	linear models	6
