# Supplementary material for: Bibliometric analysis of cardiometabolic disorders studies involving NO2, PM2.5 and noise exposure
Source: BMC Public Health. 2019 Jul 4;19:877. doi: 10.1186/s12889-019-7195-1 (PMC6610906; doi:10.1186/s12889-019-7195-1)
Supplement: Supplementary file 5 — Table S2. Difference in index keywords between cardiometabolic references involving noise exposure with H-designs and NH-designs. (PDF 86 kb) [file 12889_2019_7195_MOESM5_ESM.pdf]

S2 Table. Difference in index keyword between cardiometabolic references involving noise exposure with H-design and NH-design

	Relatively frequency		
	H-design	NH-design	Absolute difference
ambient air	.27	.14	0.13
united states	.26	.15	0.11
exhaust gas	.13	.03	0.10
systolic blood pressure	.08	.20	-0.12
diastolic blood pressure	.08	.18	-0.10
motor vehicles	.02	.09	-0.07
