# Supplementary material for: Bibliometric analysis of cardiometabolic disorders studies involving NO2, PM2.5 and noise exposure
Source: BMC Public Health. 2019 Jul 4;19:877. doi: 10.1186/s12889-019-7195-1 (PMC6610906; doi:10.1186/s12889-019-7195-1)
Supplement: Supplementary file 6 — Table S3. The top 10 cited references involving diabetes. (PDF 168 kb) [file 12889_2019_7195_MOESM6_ESM.pdf]

S3 Table. The top 10 cited references involving diabetes

Author*	Title	Publish year	Publish journal	Total number of citation
Miller K.A. et al.	Long-term exposure to air pollution and incidence of cardiovascular events in women	2007	New England Journal of Medicine	970
Beelen R. et al.	Effects of long-term exposure to air pollution on natural-cause mortality: An analysis of 22 European cohorts within the multicentre ESCAPE project	2014	The Lancet	344
O'Neil M. S. et al.	Diabetes enhances vulnerability to particulate air pollution-associated impairment in vascular reactivity and endothelial function	2005	Circulation	302
Ostro B. et al.	Fine particulate air pollution and mortality in nine California counties: Results from CALFINE	2006	Environmental Health Perspectives	257
Zanobetti A. et al.	The effect of particulate air pollution on emergency admissions for myocardial infarction: A multicity case-crossover analysis	2005	Environmental Health Perspectives	238
Zanobetti A. et al.	Fine particulate air pollution and its components in association with cause-specific emergency admissions	2009	Environmental Health: A Global Access Science Source	227
Dubowsky S.D. et al.	Diabetes, obesity, and hypertension may enhance associations between air pollution and markers of systemic inflammation	2006	Environmental Health Perspectives	226
Park S. K. et al.	Effects of air pollution on heart rate variability: The VA normative aging study	2005	Environmental Health Perspectives	202
Krämer U. et al.	Traffic-related air pollution and incident type 2 diabetes: Results from the SALIA cohort study	2010	Environmental Health Perspectives	184
Rückerl R. et al.	Air pollution and inflammation (Interleukin-6, C-reactive protein,	2007	Environmental Health Perspectives	175

	fibrinogen) in myocardial infarction survivors	

*only shows the first author
